# Comparing the effect of peritoneal dialysis cycler type on patient-reported satisfaction, support needs and treatments

**DOI:** 10.1186/s12882-022-02854-z

**Published:** 2022-06-21

**Authors:** Osama El Shamy, Sara Atallah, Shuchita Sharma, Jaime Uribarri

**Affiliations:** 1 Division of Nephrology and Hypertension, Vanderbilt University Medical Center, 1161 21st Avenue South, MCN South-3305, Nashville, TN 37232 USA; 2 Icahn School of Medicine at Mount Sinai, Division of Nephrology, New York City, NY USA

**Keywords:** Peritoneal dialysis, Telehealth, Telenephrology, Cyclers, Dialysis

## Abstract

**Background:**

Most patients on peritoneal dialysis (PD) in the United States choose automated PD via cyclers. Cyclers have evolved considerably over time with older versions (e.g. HomeChoice Pro) replaced by more sophisticated and technologically advanced versions (e.g. Amia). Understanding the effect that different cyclers and their features have on patient treatments and support needs is important.

**Methods:**

Single center study with retrospective and prospective arms. *Retrospective arm*: Patients > 18 years old, on Amia or HomeChoice Pro (HC) for ≥ 3 months between 8/1/17 and 1/31/18. Number of office/telephone encounters, PD-related emergency room visits/hospitalizations, PD training days, and dialysis adequacy (Kt/V) were recorded.

*Prospective arm*: Patients > 18 years old, on Amia or HC for ≥ 3 months between 9/1/19 and 2/29/20 were surveyed on their comfort, troubleshooting, satisfaction and reported assistance needed with their cyclers.

**Results:**

*Retrospective arm*: 43 patients on AMIA and 27 patients on HC. Number of PD training days, Kt/Vs achieved, PD-related telephone/office encounters, and PD-related emergency room visits/hospitalizations were all similar.

*Prospective Arm*: 32 patients on AMIA and 6 patients on HC. Higher rate of patient comfort with AMIA, but similar overall patient satisfaction with both cyclers. No difference in terms of patient-reported troubleshooting issues requiring assistance.

**Conclusions:**

Despite the difference in features provided between the 2 cyclers, patient overall satisfaction rates were high irrespective of the PD cycler. The HomeChoice Pro and AMIA cycler patients had a similar number of PD training days, PD-related telephone/office encounters, and PD-related emergency room visits/hospitalizations.

**Trial registration:**

This study was approved by the Icahn School of Medicine at Mount Sinai Institutional Review Board (IRB-17–02704).

**Supplementary Information:**

The online version contains supplementary material available at 10.1186/s12882-022-02854-z.

## Introduction

Most patients in the United States who opt to be on peritoneal dialysis (PD) choose automated PD, where they perform their home PD treatments using cyclers. However, patients are often not involved in deciding which PD cycler they are trained on and treated with. This is because many PD units only offer one type of cycler for their patients, and in units where there are multiple options for patients, the care team often chooses the cycler for the patient. For these reasons, patients do not switch to a different cycler unless recurrent isssues arise with their current cycler.

Cyclers have evolved considerably over time with older versions replaced by more sophisticated and technologically advanced versions. One such advancement was the introduction of the AMIA cycler by Baxter with the Sharesource remote connectivity platform. This platform helps facilitate providers’ ability to monitor and manage their home dialysis patients’ treatments remotely. With its animated graphics, touch screen panel, voice guidance, automated instructions and troubleshooting features, the AMIA system had the potential of positively affecting clinical outcomes. The AMIA system was FDA-approved in October 2015. Prior to this, most patients on APD in units contracted with Baxter in the United States were using the HomeChoice (HC) Pro cycler. This cycler does not have any of these aforementioned capabilities that AMIA provides, including the Sharesource connectivity platform. Both cyclers also have several common features such as last bag option, hydraulic flows, program volumes, as well as time and data storage.

It has been previously shown that remotely monitoring treatments of PD patients is associated with fewer admissions, shorter hospital stays and lower technique failure rates [1]. We set out to compare the effect of these two types of cyclers on the quality of dialysis delivered, number of PD training days, telephone encounters, dialysis unit office encounters, emergency room visits and hospitalizations. We also surveyed patients to determine their level of satisfaction with their respective cyclers.

## Methods

This is a single center study with retrospective (8/1/17—1/31/18) and prospective (9/1/19—2/29/20) arms. Each arm was comprised of both prevalent and incident adult patients (> 18 years old) who were on PD for ≥ 3 months during the study period at the Mount Sinai Home Dialysis Unit. For the retrospective arm, the number of office (excluding patients’ regular monthly visit) and telephone encounters to the dialysis unit, as well as PD-related emergency room visits and hospitalizations were obtained using the electronic medical record system. We then looked back at the total number of PD training days (up to 8 h per day) required for patients when they were initiating PD. In our dialysis unit, patients usually undergo both continuous ambulatory PD (CAPD) and continuous cycling peritoneal dialysis (CCPD) training before initiating dialysis. The total number of days of PD training were documented. Patients who underwent CAPD training more than 1 month prior to CCPD training were excluded as the authors hypothesize that this would decrease the number of training days required for CCPD since patients were already familiar with PD at that point. Dialysis adequacy (total Kt/V) values during the study period were obtained using the Baxter’s Adequest program.

Patients in the prospective arm were asked to complete a short survey in which they answered questions about their level of comfort, troubleshooting and setting adjustment capabilities, as well as their satisfaction with their current cycler. For each question, patients rated their response on a scale of 1 (Strongly Disagree) to 5 (Strongly Agree). Moreover, patients were asked to report the number of times they needed to call either Baxter or the dialysis unit for questions or concerns about their cycler in the preceding month with the options being 1) Zero, 2) 1–2 times, 3) 3–4 times, and 4) More than 4 times. Cycler-related issues included cyclers not turning on, cycler reprogramming, ultrafiltration deviation alarms, lost dwell time, cassette failure, system error messages, and low flow alarms.

Please see supplemental section S1 for complete survey questions and answer choices.

Statistical analysis was performed using the unpaired t-test and z-test to determine statistical significance between patients on AMIA and those on HC.

This study was approved by the Icahn School of Medicine at Mount Sinai Institutional Review Board (IRB-17–02,704) and all methods were carried out in accordance with the guidelines and regulations.

## Results

### Retrospective cohort

Between 8/1/17 and 1/31/18, 43 patients were using the AMIA cycler and 27 patients were using the HC cycler. Patients on the AMIA cycler were older than their HC counterparts (58.6 vs 53.5 years, *p* = 0.18), with a larger proportion of African American and Asian patients (Table 1).

**Table 1:** Demographics and outcomes of patients in the retrospective cohort

	**AMIA**	**HomeChoice**	***p*-value**
Number of patients	43	27	
Age (years)	58.6 ± 14.5	53.5 ± 16.3	0.18
**Race:**			
African American	21 (49%)	9 (33%)	0.20
White	8 (19%)	8 (30%)	0.28
Asian	6 (14%)	2 (7%)	0.40
**Ethnicity:**			
Hispanic	7 (16%)	8 (30%)	0.18
Number of PD training days	6.3	6.4	0.93
Time on PD prior to study (months)	17 (IQR: 4 -23)	51 (IQR: 25–64)	< 0.001
**Rate of PD-related encounters:**			
Telephone and Office	1.6	1.4	0.81
ED and Hospitalization	0.3	0.3	1.00
Cycler issues	0.09	0.15	0.49

During the study period, patients in the AMIA and HC groups were on PD for similar time periods, an average of 5.7 and 5.4 months, respectively. Prior to the study period, the AMIA group had been on PD for a shorter period of time than their HC counterparts (17 vs 51 months, *p* < 0.001). The average number of PD training days per patient were similar (6.3 vs 6.4, *p* = 0.929), and so was the dialysis adequacy (Kt/V) achieved (1.92 vs 1.93, *p* = 0.829).

Patients on AMIA had a total of 33 office and telephone encounters during the study period (outside of the regular monthly office visit): 12 peritonitis, 4 exit site issues, 6 cycler-related issues, 3 effluent-related issues (hazy/fibrin/bloody), 2 volume overload, 2 blood pressure issues, 4 abdominal pain/discomfort (not peritonitis). These patients also had a total of 4 emergency room visits and hospitalizations: 1 volume overload, 2 peritonitis and 1 cycler malfunction.

On the other hand, patients on HC had a total of 18 office and telephone encounters during the study period (outside of the regular monthly office visit): 7 peritonitis, 3 exite site issues, and 8 cycler-related issues. These patients also had a total of 4 emergency room visits and hospitalizations: 3 peritonitis and 1 blood pressure-related.

Statistical analysis demonstrated no statistically significant difference between the two group in terms of office and telephone encounters (33/43 vs 18/27, *p* = 0.36) and emergency room vists and hospitalizations (4/43 vs 4/27, *p* = 0.48).

Patients on the AMIA cycler had a lower rate of cycler-related issues than their HC counterparts (0.28 vs 0.59 issues/patient/year, *p* = 0.008).

### Prospective cohort

Between 9/1/19 and 2/29/20, 32 patients (5 incident, 27 prevalent) using the AMIA cycler and 6 patients (all prevalent) using the HC cycler consented for the study. Patient demographics are shown in Table 2.

**Table 2:** Demographics and outcomes of patients in the prospective cohort

	**AMIA**	**HC**	
Number of patients	32	6	
Age (years)	53.3 ± 14.4	54.3 ± 9.9	
**Race:**			
African American	14 (44%)	0 (0%)	
White	5 (16%)	1 (17%)	
Asian	4 (13%)	1 (17%)	
Other	0 (0%)	4 (67%)	
**Ethnicity:**			
Hispanic	9 (28%)	4 (67%)	
**Survey Results:**	AMIA (Incident)	AMIA (Prevalent)	HC (Prevalent)
Comfort with the cycler	4.8	4.9	4.5
Overall Satisfaction	4.6	4.8	4.5
Assistance required	1.2	1.2	1.5

On a scale of 1–5, when assessing patients’ level of comfort, providers’ ability to troubleshoot and change their settings, and satisfaction with the cycler, both the prevalent and incident patients on AMIA responded highly favorably (4.9 vs 4.8, 4.9 vs 4.6 and 4.8 vs 4.6).

Comparing the prevalent AMIA to prevalent HC patients’, patients on AMIA reported higher levels of comfort (4.9 vs 4.5). However, overall satisfaction with the cycler was similar between the two groups. When asked how many times in the previous month they needed to contact either Baxter or the dialysis unit for cycler-specific issues, the mean response for incident AMIA and prevalent AMIA patients were the same at 1.2. There was no difference between prevalent AMIA and HC patients.

## Discussion

Two of the several APD options that patients on PD in the United States have are the AMIA and HomeChoice Pro cyclers, both of which are provided in our home dialysis unit. Features that the technologically advanced AMIA cycler provides that are not present in the HomeChoice Pro cycler include animated graphics, touch screen panel, voice guidance, automated instructions and troubleshooting features as well as remote monitoring through Sharesource. The fact that we found that patients on AMIA – despite their shorter PD vintage – had a lower rate of cycler-related issues compared to their HC counterparts, could be testament to the positive effect that these features have on patients’ hands-on troubleshooting and experience with the cycler. However, despite these capabilities, we found no statistically significant difference between it and the HC cycler in terms of PD-related telephone and office encounters, emergency department visits and hospitalizations.

Our findings differ from a previous study by Sanabria et al [2] where after matching remote patient monitoring (RPM) patients with their non-RPM counterparts, they found significantly lower hospitalization rates and number of in-patient hospital days in the RPM group. There was no reason to suspect that there would be any difference in dialysis adequacy achieved in the two groups, and we found it to be similar as was previously shown [3].

In our retrospective cohort, we found that the number of PD training days required were similar between the two groups. This is in contrast to our previous finding that patients being trained on the AMIA cycler required 33% less time than their HC counterparts [3]. A potential confounder in our study is that the HC patients were younger (although not statistically significant) and had been in PD for much longer than their AMIA counterparts. Moreover, the length of PD training required depends on the individual patient and staff member doing the training. The dialysis unit nursing staff performing the patient PD training was the same during the retrospective period.

In the prospective arm, few patients were on HC during the study period – all of which were prevalent patients. For this reason, we chose to focus on surveying those patients, rather than obtaining the same data that we did for the retrospective arm. We found that despite prevalent AMIA patients reporting higher levels of comfort with their cycler, patients’ overall satisfaction was similar for both prevalent AMIA and HC patients. There was also no difference between these two groups in terms of patient-reported troubleshooting issues requiring assistance.

Creating technically sophisticated cyclers may come at the cost of increased cycler-related issues due to glitches in technology and loss of simplicity. This is something that we did not find to be the case in patients’ reported ability to troubleshoot, change settings of and overall satisfaction with the AMIA cycler.

From a clinician’s perspective, the importance of remote patient treatment monitoring should not be undermined. Daily telemonitoring of home dialysis patients has been previously shown to be cost-effective, allows early detection and resolution of issues, improving dialysis compliance and patients’ quality of life [4]. In this study, we did not specifically explore the effect of remote monitoring on our patients’ outcomes. The experiences of providers across the world and the importance of telenephrology with remote monitoring in caring for their PD patients was also demonstrated during the COVID-19 pandemic [5–7]. These benefits, of course, do come with challenges. Data security, reduced staff contact, liability associated with delayed review of alarms, absorbing the higher cost, and the acceptance of technology are amongst the challenges that face providers and healthcare systems when incorporating this technology into common practice [8].

Our study has limitations, the most important of which is the small number of participants. The goal of the retrospective cohort was to examine a time period in which the number of patients on AMIA and HC were similar. With most of our new start PD patients using the AMIA cycler since its FDA-approval in 2015, it was expected that the patients’ time on PD prior to the study period in the HC group would be greater than AMIA. Patients’ time on the cycler increases their familiarity with the nuances of the cycler and their level of comfort in troubleshooting any alarms or issues that may arise. This would skew the data in favor of patients on HC. Given that over 85% of the patients in our home dialysis unit use the AMIA cycler nowadays, it was difficult to obtain a comparable number of patients on HC in the prospective cohort, the best way to clearly compare both therapies. Another confounder is that documentation of the number of telephone encounters to the dialysis unit in our data collection did not include direct patients support calls to Baxter technical support line as we did not have access to this information. Instead, we relied on patient recollection to obtain this information.

A direct comparison of AMIA to HC would require a large cohort of patients who have experience with both cyclers for a prolonged period of time, allowing for the assessment of their clinical outcomes, reported experiences and support needs. Unfortunately, this is something that is difficult to achieve. Of note, this study was conducted prior to the FDA’s approval of HomeChoice Claria in November 2020 – a version of the HC cycler which provides connectivity to the Sharesource platform.

As technological advancements continue to drive our society and medicine forward, so too will the push for their implementation to help maximize patient care. The role of remote monitoring in producing favorable patient outcomes has been previously shown and is something that will continue to be studied closely. Moreover, the coronavirus disease of 2019 (COVID-19) has necessitated further expansion of telehealth services in order to help facilitate social distancing. This was especially relevant for patients on home dialysis modalities who come to the clinic for their monthly visits and assistance with dialysis-related issues [5–7].

We were not able to demonstrate a statistically significant difference between the AMIA and HC cyclers in terms of patient overall satisfaction, PD training days, PD-related telephone/office encounters, and PD-related emergency room visits/hospitalizations. These findings demonstrate that our patients adapt to the life-changing event that is PD initiation similarly, irrespective of the PD cycler they are initiated on.

## Supplementary Information

**Additional file 1:** **S1.** Patient/Caregiver Amia Survey. **S2. **Patient/Caregiver HomeChoice Pro Survey.

**Additional file 2.** Amia vs HomeChoice Prospective Data.

**Additional file 3.** Amia vs HomeChoice Retrospective Data. 

## Data Availability

The datasets used and/or analysed during the current study are available from the corresponding author on reasonable request.
