# Supplementary material for: Comparing the effect of peritoneal dialysis cycler type on patient-reported satisfaction, support needs and treatments
Source: BMC Nephrol. 2022 Jun 21;23:217. doi: 10.1186/s12882-022-02854-z (PMC9209826; doi:10.1186/s12882-022-02854-z)
Supplement: Supplementary file 1 — Additional file 1: S1. Patient/Caregiver Amia Survey. S2. Patient/Caregiver HomeChoice Pro Survey. [file 12882_2022_2854_MOESM1_ESM.docx]

Supplemental Section.

S1. Patient/Caregiver Amia Survey

Please rate the following statements to the best of your ability.

1. I feel comfortable managing the Amia PD cycler at home

| [ ] Strongly Disagree | [ ] Disagree | [ ] Neutral | [ ] Agree | [ ] Strongly Agree |
| --- | --- | --- | --- | --- |

1. I like that my healthcare provider can troubleshoot/change the settings from the clinic with the Amia PD cycler

| [ ] Strongly Disagree | [ ] Disagree | [ ] Neutral | [ ] Agree | [ ] Strongly Agree |
| --- | --- | --- | --- | --- |

1. I am satisfied with the Amia PD cycler system that I use at home for my peritoneal dialysis

| [ ] Strongly Disagree | [ ] Disagree | [ ] Neutral | [ ] Agree | [ ] Strongly Agree |
| --- | --- | --- | --- | --- |

1. How many times in the last month did you need help from your healthcare provider with your Amia PD cycler (troubleshooting/changing settings)?

| [ ] Zero | [ ] 1-2 times | [ ] 3-4 times | [ ] More than 4 times |
| --- | --- | --- | --- |

Other comments about the Amia PD cycler system:

Thank you for taking the time to complete the survey!

S2. Patient/Caregiver HomeChoice Pro Survey

Please rate the following statements to the best of your ability.

1. I feel comfortable managing the HomeChoice PD cycler at home

| [ ] Strongly Disagree | [ ] Disagree | [ ] Neutral | [ ] Agree | [ ] Strongly Agree |
| --- | --- | --- | --- | --- |

1. I would like it if my healthcare provider could troubleshoot/change the settings from the clinic with the HomeChoice PD cycler

| [ ] Strongly Disagree | [ ] Disagree | [ ] Neutral | [ ] Agree | [ ] Strongly Agree |
| --- | --- | --- | --- | --- |

1. I am satisfied with the HomeChoice PD cycler system that I use at home for my peritoneal dialysis

| [ ] Strongly Disagree | [ ] Disagree | [ ] Neutral | [ ] Agree | [ ] Strongly Agree |
| --- | --- | --- | --- | --- |

1. How many times in the last month did you need help from your healthcare provider with your HomeChoice PD cycler (troubleshooting/changing settings)?

| [ ] Zero | [ ] 1-2 times | [ ] 3-4 times | [ ] More than 4 times |
| --- | --- | --- | --- |

Other comments about the HomeChoice PD cycler system:

Thank you for taking the time to complete the survey!
